# Supplementary material for: A miniaturized MR1 metabolite display system with native-like protein features
Source: bioRxiv. 2026 Apr 15:2026.04.13.718121. Preprint. [Version 1] doi: 10.64898/2026.04.13.718121 (PMC13104969; doi:10.64898/2026.04.13.718121)
Supplement: Supplement 1 [file NIHPP2026.04.13.718121v1-supplement-1.pdf]

bioRxiv preprint doi: https://doi.org/10.64898/2026.04.13.718121; this version posted April 15, 2026. The copyright holder for this preprint (which was not certified by peer review) is the author/funder, who has granted bioRxiv a license to display the preprint in perpetuity. It is made available under aCC-BY 4.0 International license.

Supporting Information

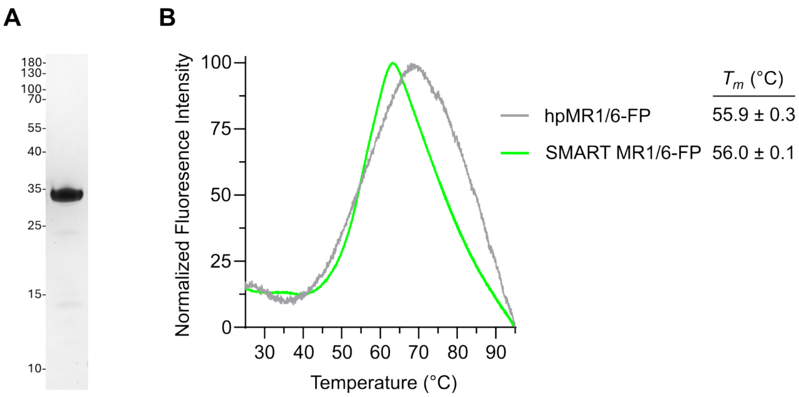

Figure S1. Thermal stability of SMART-MR1 compared to full-length MR1.
A. SDS-PAGE gel of purified SMART-MR1 refolded with 6-FP. **B.** Normalized DSF traces of purified SMART-MR1 and human platform MR1 (hpMR1) refolded with 6-FP. Data are mean ± s.d. for $n = 3$ technical replicates.

bioRxiv preprint doi: https://doi.org/10.64898/2026.04.13.718121; this version posted April 15, 2026. The copyright holder for this preprint (which was not certified by peer review) is the author/funder, who has granted bioRxiv a license to display the preprint in perpetuity. It is made available under aCC-BY 4.0 International license.

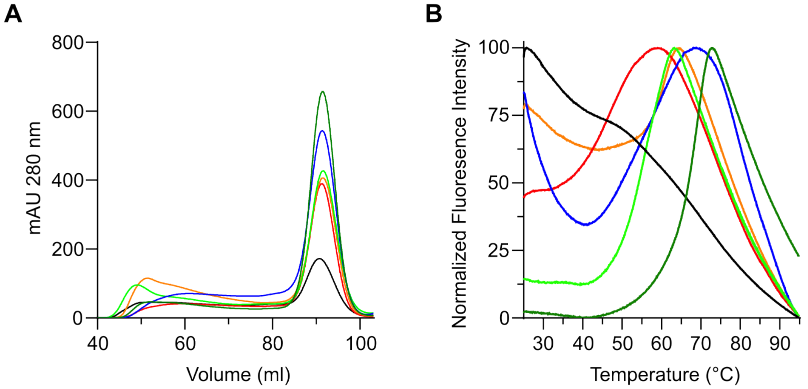

C

	Yield (mg)	T_m (°C)
Empty	1.81	N.D.
Ac-6-FP	6.45	67.1 ± 0.1
5FSA	2.49	59.8 ± 0.1
6-FP	4.17	56.0 ± 0.1
5-OP-RU	4.38	55.8 ± 0.2
3FSA	2.75	46.5 ± 0.2

Figure S2. SEC traces and DSF curves of refolded SMART-MR1 complexes.
A. Size exclusion chromatography (SEC) traces of SMART-MR1 refolded in the absence (empty) and presence of ligand. SEC traces for MR1 are color coded as shown in panel C. **B.** Normalized DSF traces of purified empty and ligand loaded SMART MR1. **C.** Summary of refolding yields obtained from SEC experiments and melting temperatures (T_m) obtained from DSF experiments. Data are mean ± s.d. for n = 3 technical replicates.

bioRxiv preprint doi: https://doi.org/10.64898/2026.04.13.718121; this version posted April 15, 2026. The copyright holder for this preprint (which was not certified by peer review) is the author/funder, who has granted bioRxiv a license to display the preprint in perpetuity. It is made available under aCC-BY 4.0 International license.

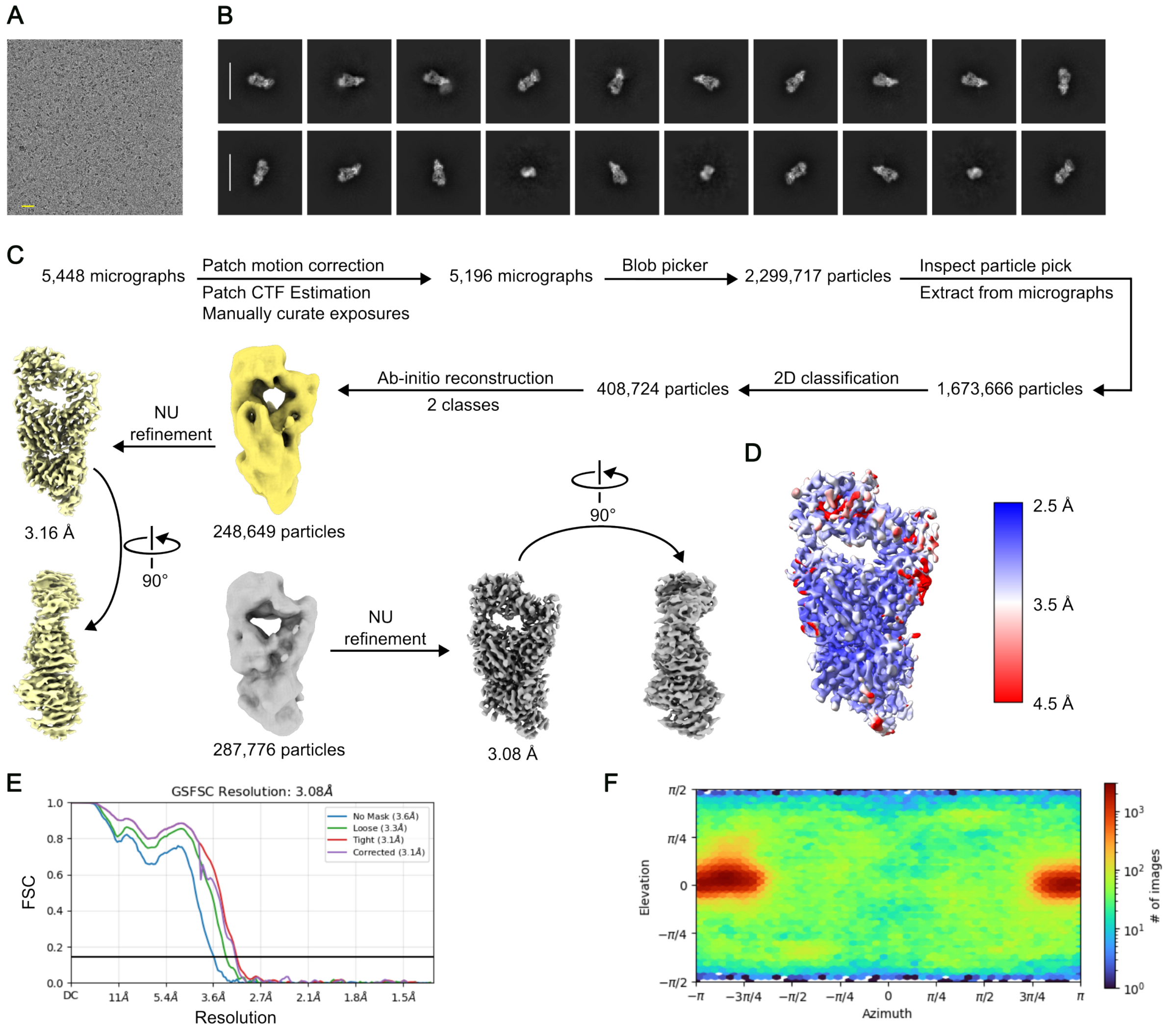

Figure S3. Cryo-EM data processing for A-F7 TCR in complex with SMART-MR1-5-OP-RU.

A. Representative micrograph of the collection for A-F7 TCR/SMART-MR1-5-OP-RU complex. Scale bar in yellow is 200 Å. **B**. Representative 2D classes of A-F7 TCR/SMART-MR1-5-OP-RU complex. Scale bar in white is 150 Å. **C**. Cryo-EM data processing workflow of A-F7 TCR/SMART-MR1-5-OP-RU complex. **D**. Local-resolution estimation of reconstructed map as determined within CryoSPARC. **E**. Gold-standard FSC curves used for global-resolution estimates within CryoSPARC. **F**. Viewing direction distribution of the reconstructed map.

bioRxiv preprint doi: https://doi.org/10.64898/2026.04.13.718121; this version posted April 15, 2026. The copyright holder for this preprint (which was not certified by peer review) is the author/funder, who has granted bioRxiv a license to display the preprint in perpetuity. It is made available under aCC-BY 4.0 International license.

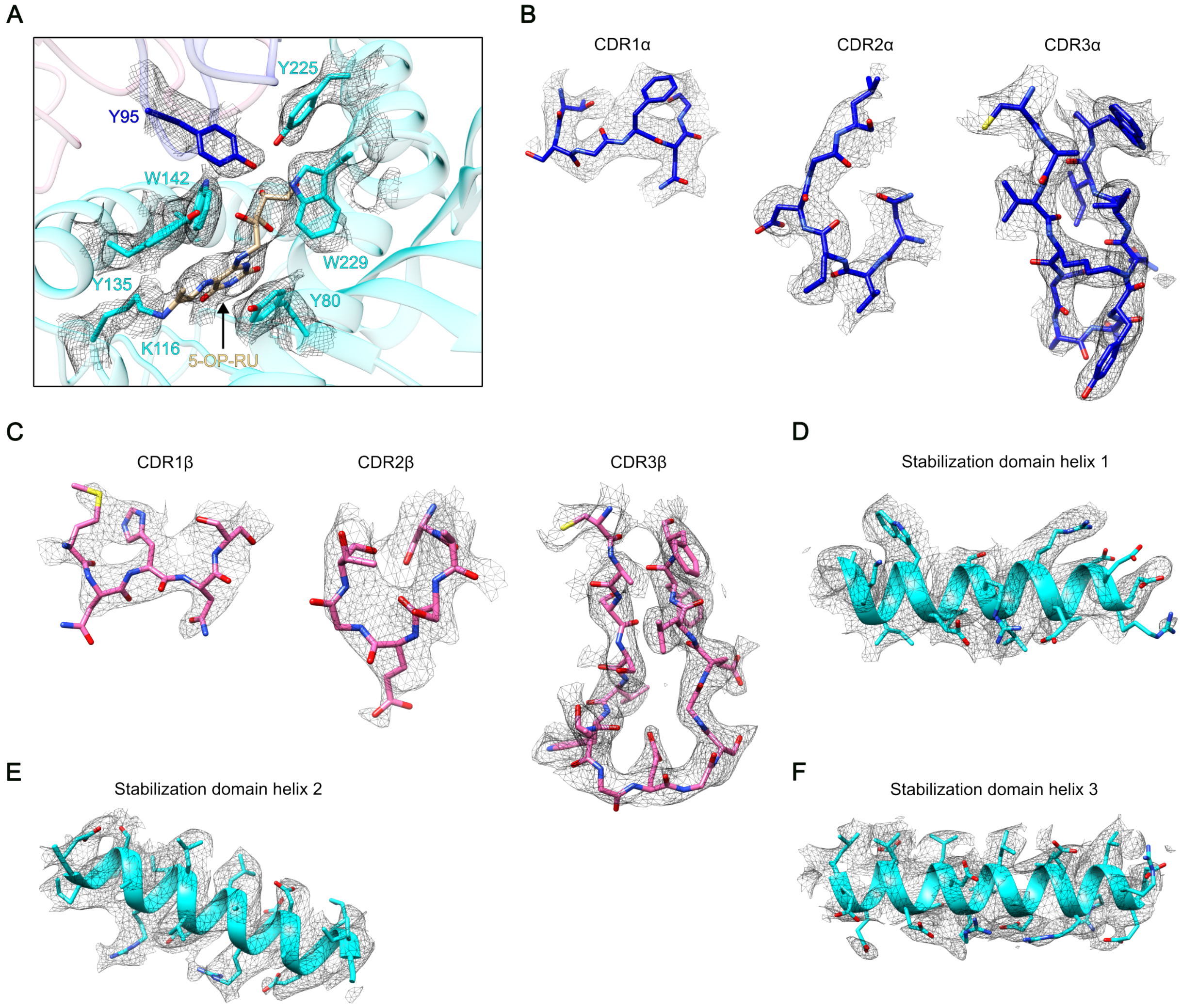

Figure S4. Quality of SMART-MR1-A-F7 cryo-EM map.

A. Cryo-EM density of 5-OP-RU and the residues interacting with it. MHC is colored cyan, TCRα in blue, TCRβ in pink, and 5-OP-RU in light brown. Map is contoured at 0.196. **B.** Cryo-EM densities of CDRs of TCRα. Maps are contoured at 0.150 (CDR1α), 0.200 (CDR2α), and 0.180 (CDR3α). **C.** Cryo-EM densities of CDRs of TCRβ. Maps are contoured at 0.240 (CDR1β), 0.140 (CDR2β), and 0.120 (CDR3β). **D-F.** Cryo-EM densities of stabilization domain helices. Maps are contoured at 0.095 (D), 0.085 (E), and 0.070 (F).

bioRxiv preprint doi: https://doi.org/10.64898/2026.04.13.718121; this version posted April 15, 2026. The copyright holder for this preprint (which was not certified by peer review) is the author/funder, who has granted bioRxiv a license to display the preprint in perpetuity. It is made available under aCC-BY 4.0 International license.

Supplementary Table S1. Cryo-EM data collection, refinement and validation statistics SMART-MR1/A-F7 TCR

	Human MAIT A-F7 TCR in complex with miniaturized MR1-5-OP-RU<br>(EMDB EMD-75491)<br>(PDB 10VM)
**Data collection and processing**	
Magnification	165,000×
Voltage (kV)	200
Electron exposure (e–/Å^2)	40
Defocus range (μm)	-0.8 to -2.0
Pixel size (Å)	0.6975
Symmetry imposed	C1
Initial particle images (no.)	2,299,717
Final particle images (no.)	287,776
Map resolution (Å)	3.08
FSC threshold	0.143
Map resolution range (Å)	2.58-3.58
**Refinement**	
Initial model used (PDB code)	6PUC, 9NDS
Model resolution (Å)	3.3
FSC threshold	0.5
Model resolution range (Å)	3.0-3.3
Model composition	
Non-hydrogen atoms	5442
Protein residues	680
Ligands	1
B factors (Å^2)	
Protein (min/max/mean)	14.82/135.30/60.82<br>10.56/34.03/23.29
Ligand (min/max/mean)	
R.m.s. deviations	
Bond lengths (Å)	0.004

bioRxiv preprint doi: https://doi.org/10.64898/2026.04.13.718121; this version posted April 15, 2026. The copyright holder for this preprint (which was not certified by peer review) is the author/funder, who has granted bioRxiv a license to display the preprint in perpetuity. It is made available under aCC-BY 4.0 International license.

Bond angles (°)	0.605
Validation	
MolProbity score	2.67
Clashscore	12.55
Poor rotamers (%)	6.07
Ramachandran plot	
Favored (%)	92.56
Allowed (%)	7.44
Disallowed (%)	0.00
